# Toxicogenomic analysis of susceptibility to inhaled urban particulate matter in mice with chronic lung inflammation

**DOI:** 10.1186/1743-8977-6-6

**Published:** 2009-03-11

**Authors:** Errol M Thomson, Andrew Williams, Carole L Yauk, Renaud Vincent

**Affiliations:** 1 Environment and Occupational Toxicology Division, Environmental Health Science and Research Bureau, Health Canada, Ottawa, Canada; 2 Biostatistics and Epidemiology Division, Environmental Health Science and Research Bureau, Health Canada, Ottawa, Canada; 3 Department of Biochemistry, Microbiology & Immunology, University of Ottawa, Ottawa, Canada

## Abstract

**Background:**

Individuals with chronic lung disease are at increased risk of adverse health effects from airborne particulate matter. Characterization of underlying pollutant-phenotype interactions may require comprehensive strategies. Here, a toxicogenomic approach was used to investigate how inflammation modifies the pulmonary response to urban particulate matter.

**Results:**

Transgenic mice with constitutive pulmonary overexpression of tumour necrosis factor (TNF)-α under the control of the surfactant protein C promoter and wildtype littermates (C57BL/6 background) were exposed by inhalation for 4 h to particulate matter (0 or 42 mg/m^3 ^EHC-6802) and euthanized 0 or 24 h post-exposure. The low alveolar dose of particles (16 μg) did not provoke an inflammatory response in the lungs of wildtype mice, nor exacerbate the chronic inflammation in TNF animals. Real-time PCR confirmed particle-dependent increases of CYP1A1 (30–100%), endothelin-1 (20–40%), and metallothionein-II (20–40%) mRNA in wildtype and TNF mice (p < 0.05), validating delivery of a biologically-effective dose. Despite detection of striking genotype-related differences, including activation of immune and inflammatory pathways consistent with the TNF-induced pathology, and time-related effects attributable to stress from nose-only exposure, microarray analysis failed to identify effects of the inhaled particles. Remarkably, the presence of chronic inflammation did not measurably amplify the transcriptional response to particulate matter.

**Conclusion:**

Our data support the hypothesis that health effects of acute exposure to urban particles are dominated by activation of specific physiological response cascades rather than widespread changes in gene expression.

## Background

Increased levels of particulate matter (PM) are associated with respiratory and cardiovascular morbidity and mortality [1-3]. Acute adverse health effects associated with PM are generally attributed to susceptible subgroups of the population, including individuals with respiratory diseases such as chronic obstructive pulmonary disease (COPD) [4-7]. Exacerbation of existing inflammation and increased generation of oxidative stress provoking an acute phase response is one mechanism through which PM may cause adverse cardiovascular effects [8]. Inhaled particles aggravate existing epithelial lesions in the lungs [9], and impairment of epithelial barrier function can result in increased translocation of particles to the interstitium [10]. Once in the interstitium, particles are less likely to be cleared via macrophage phagocytosis, and can cause interstitial inflammation, induce direct effects on local cell populations (including macrophages, fibroblasts, endothelial cells, and neutrophils) and drain to local lymph nodes [11,12]. Chronic inflammation and oxidative stress may also prime the lungs to respond to particles with increased production of reactive oxygen species and inflammatory mediators, exacerbating the existing disease state [13]. In addition, inflammation and tissue damage may facilitate direct interaction of PM factors with the pulmonary endothelium, disturbing vascular function [14,15] and increasing the risk of acute cardiac events from plaque instability or reduced myocardial perfusion. Investigating the interaction of inhaled particles and host factors governing susceptibility may be critical to understanding acute health effects related to PM exposure.

Urban PM is a heterogeneous mixture of organic and inorganic compounds likely to provoke a number of transcriptional and physiological responses in lung tissue [16,17]. We have previously established that mRNA and plasma levels of the potent vasoconstrictor endothelin (ET)-1 are increased 25–50% after particle inhalation, a response that does not require acute lung injury [9,14,18-20]. Increases of plasma ET-1 in this range have high predictive value for chronic heart failure [21], indicating that the magnitude of ET-1 change need not be large to have pathophysiological relevance. Substantiating these animal studies, elevated plasma ET-1 and increased mean pulmonary arterial pressure have been measured in children exposed to high pollutant levels in Mexico City [22]. While lung injury may not be a prerequisite for pathway activation, existing injury and inflammation can exacerbate or alter the response to inhaled contaminants [9,19]. Interaction of the physiological state of the lungs and the physicochemical properties of inhaled particles likely involves multiple pathways, and such complexity is difficult to investigate by conventional means. Genomic approaches can contribute to the elucidation of mechanisms by providing context for defined toxicant-related changes and by identifying novel toxicant-responsive pathways.

Few studies have examined responses of the pulmonary transcriptome to urban PM. In an intratracheal instillation study, lungs of spontaneously hypertensive male rats exposed to the urban PM preparation EHC-93 exhibited differential expression of 132 genes with fold-changes of at least 1.5-fold 2–6 h post-exposure, including genes involved in oxidative stress, inflammatory response, and transcription, with fewer genes affected 15–21 h and 24–40 h post-exposure [23]. As samples were pooled for each time point it was not possible to assess the statistical significance of these effects. A subchronic inhalation exposure study of ApoE/low-density-lipoprotein double knockout mice exposed to concentrated ambient PM 6 h/day, 5 d/wk, for 4 mo did not identify any differentially expressed genes in lung tissue 3–4 d after the final exposure according to statistical analysis of results, although 95 probes were up- or down-regulated by at least 1.5-fold [24]. A macroarray study with 1176 probes found little differential expression (9 genes) 3 d after intratracheal instillation of 10 mg Cardiff PM, and there was poor agreement with PCR data [25]. To date, no microarray study has examined immediate or early effects of inhaled urban PM on pulmonary gene expression. Because of the rapid onset of cardiovascular effects detected in epidemiologic work [26,27], and the early response of endothelin system genes in previous targeted gene expression studies [19,28], this is an important window for investigation.

In the present study we used a toxicogenomic approach to investigate how chronic inflammation modifies the transcriptional response of the lungs to inhaled urban PM. We hypothesized that a transcriptome-wide approach would allow detection of effects not investigated by conventional means. Furthermore, we hypothesized that chronic inflammation would amplify the pulmonary transcriptional response to inhaled PM. Transgenic mice with constitutive pulmonary expression of tumour necrosis factor (TNF)-α under the control of the surfactant protein (SP)-C promoter develop lung inflammation, characterized by influx of macrophages, lymphocytes, and neutrophils, alveolar disruption and airspace enlargement [29,30]. The resulting lung pathology has been well-characterized through imaging, histology, and functional analysis [29-32], and the mice have been used for assessment of effects of repeated exposure to PM and ozone [33]. Using commercial microarrays we assessed expression of roughly 21,000 transcripts in TNF mice and their wildtype (WT) littermates exposed by inhalation to urban PM or air. In this study, the combination of a relatively large sample size (n = 5/group, 40 arrays total), paired with a standard reference design controlling for confounding variables, provided a robust approach to examine transcriptional profiles associated with PM inhalation in normal and inflamed lungs. Our objectives were: 1) to evaluate the utility of microarrays to investigate pollutant-phenotype interactions at a relevant internal dose of PM; 2) to identify genes and pathways that respond to inhaled PM; 3) to investigate how inflammation modifies this response; and 4) to identify biomarkers of exposure that are robust to the physiological status of the lungs.

## Results

### Deposition modelling

**Figure 1:**
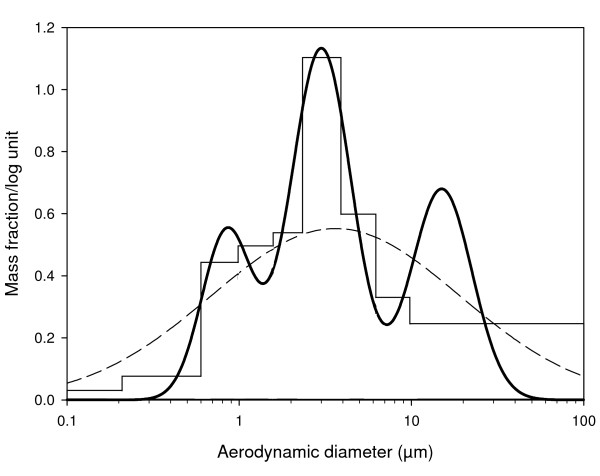
**Particle size distribution of EHC-6802**. The histogram represents the mass of particles collected on the individual cascade impactor plates. The dotted line represents the particle size distribution assuming a single mode. The dark curving line represents the multimodal fit.

EHC-6802 particles had a count median diameter (optical) of 0.58 μm. Analysis of cascade impactor data revealed a multimodal particle size distribution, with two respirable modes at 0.85 μm (aerodynamic diameter, D_AE_) and 3.0 μm D_AE_, and a non-respirable mode at 15 μm D_AE _(Figure 1). Using RDDR2 modelling software, the pulmonary compartment deposition efficiency was estimated at 11% of inhaled mass for the 0.85 μm D_AE _particle mode (20% of aerosol mass), 5% of inhaled mass for the 3.0 μm D_AE _particle mode (50% of aerosol mass), and 0.1% of inhaled mass for the 15 μm D_AE _particle mode (30% of aerosol mass). Given a concentration of 42 mg/m^3 ^and an inhaled air volume of 8 L over the 4 h exposure (minute ventilation 34 mL/min), the particle dose delivered to the pulmonary compartment was estimated at 16 μg (42 μg/L × 8L × {[0.11 × 0.20] + [0.05 × 0.50] + [0.001 × 0.30]}) on 500 cm^2 ^of alveolar septum, or 32 ng/cm^2 ^alveolar surface area. Note that the estimated dose is for normal mouse lungs. Lung parenchymal surface area decreases in adult TNF mice due to emphysema, and the ratio of parenchymal surface area between WT and TNF mice has been reported to be roughly 2:1 [30]. We have confirmed a 2:1 ratio of parenchymal surface area in our animals by stereology on paraffin sections (data not shown). The dose estimate for TNF mice is therefore 16 μg on 250 cm^2^, or 64 ng/cm^2^.

### Lung histology and differential cell counts

**Figure 2:**
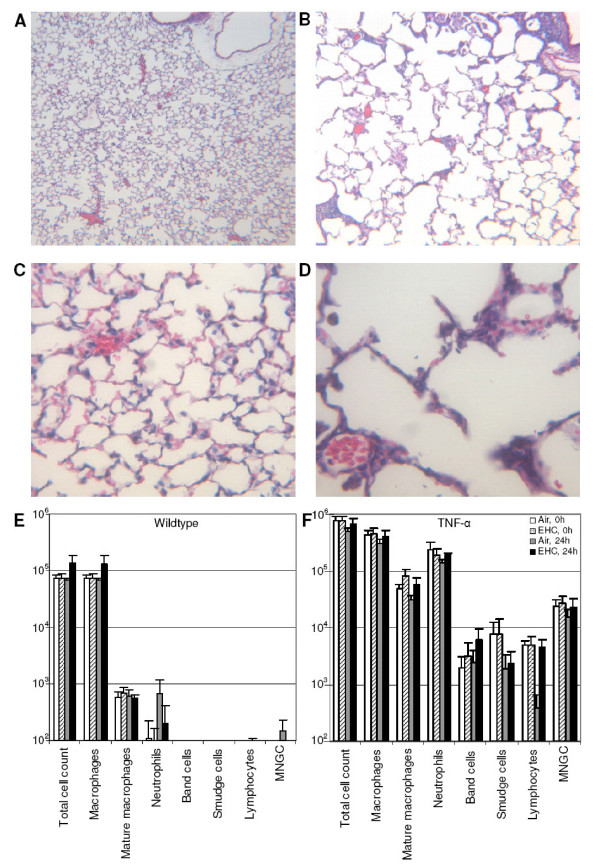
**Comparison of lung phenotype of wildtype (WT) and SP-C/TNF-α (TNF) mice**. (A-D) Mice were euthanized and lungs were inflated to 25 cm H_2_O static pressure by intratracheal instillation of 4% paraformaldehyde in PBS. Sections from WT (A, C) and TNF (B, D) mice were stained with hematoxylin and eosin. (A) WT lungs (original magnification 40×). (B) TNF lungs exhibited chronic inflammation and air-space enlargement (original magnification 40×). Comparison of WT lungs at higher magnification (C) to TNF lungs (D) revealed thickened interstitial areas (original magnification 200×). (E) Effects of PM inhalation on lavage cytology. Cells were recovered by bronchoalveolar lavage of mice exposed to EHC-6802 (0, 42 mg/m^3^) and euthanized 0 or 24 h post-exposure. Numbers of all cell types were significantly increased in TNF mice relative to their WT littermates (*Genotype *main effect, p < 0.001). There were no significant effects of particle exposure within each genotype. MNGC, multi-nucleated giant cells.

Lung histology confirmed that the TNF mice (Figure 2b, d) had chronic inflammation, enlarged alveolar spaces, and septal destruction compared to wildtype mice (Figure 2a, c). Differential counts of cells recovered by bronchoalveolar lavage confirmed greater numbers of all cell types in TNF mice relative to their WT littermates (*Genotype *main effect, p < 0.001; Figure 2ef). Cellular changes in TNF mice consisted of a 10-fold increase in cell recovery in lavage fluid, with a 5-fold increase of macrophages, pronounced neutrophilia, and the presence of lymphocytes and multinucleated giant cells. Inhalation exposure to particles for 4 h did not significantly affect cell number or composition in either WT or TNF animals, indicating that changes in gene expression resulting from PM exposure should reflect changes within the lung tissue rather than import of signal by influx of inflammatory cells.

### Confirmation of PM effects on key biological pathways

**Figure 3:**
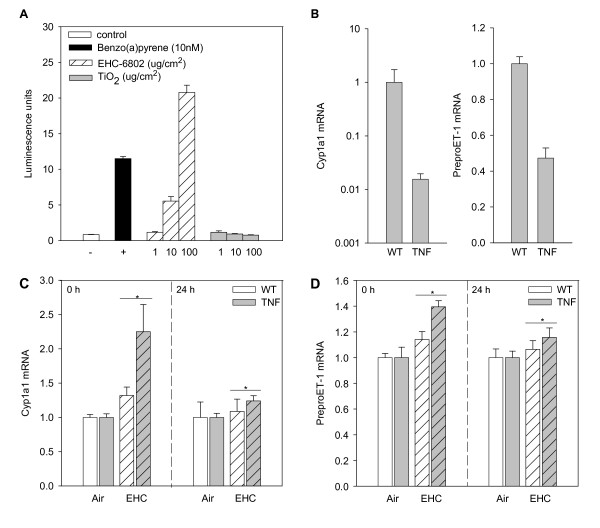
**In vitro and in vivo assessment of induction of biologically relevant pathways by EHC-6802**. (A) Aryl hydrocarbon receptor activation in vitro. H1L1.1c2 cells were exposed to vehicle, benzo(a)pyrene, EHC-6802 particles, and TiO_2_, and luciferase activity was determined. Values represent the mean ± SEM of triplicate determinations. (B) Relative basal mRNA levels of CYP1A1 and preproET-1 in the lungs of unexposed WT and TNF mice as measured by real-time PCR. Results are expressed as geometric mean ± SD (n = 4 animals/group). (C) PM effects on CYP1A1 mRNA levels in vivo. SP-C/TNF-α (TNF) mice and their wildtype (WT) littermates were exposed to 0 or 42 mg/m^3 ^EHC-6802 and euthanized 0 or 24 h post-exposure. Real-time PCR was used to determine expression. Results are expressed as geometric mean ± SD (n = 5 animals/group). *Treatment main effect, p = 0.05, one-tailed. (D) PreproET-1 mRNA levels in WT and TNF mice 0 and 24 h after exposure to EHC-6802. *Treatment main effect, p = 0.04, one-tailed.

EHC-6802 (but not TiO_2_) caused a dose-dependent increase of dioxin response element regulated luciferase activity in H1L1.1c2 cells (Figure 3a), confirming AhR-activation by these particles. To verify delivery of a biologically-effective dose of particles to the lungs, real-time PCR was used to assess the effect of EHC-6802 on pulmonary expression of two key transcripts induced by PM: the AhR-regulated gene CYP1A1 [34,35], and endothelin-1 [14,18,19]. Basal CYP1A1 mRNA levels were 70-fold lower in TNF mice (Figure 3b), in line with known effects of inflammation on P450 genes [36]. PreproET-1 mRNA levels in TNF mice were half of those in WT mice (Figure 3b), consistent with the 50% decrease of parenchymal surface area and thus 50% loss of endothelial capillary surface area. The deposition of urban particles in the lungs increased CYP1A1 mRNA (three-way ANOVA, *Treatment *main effect, p = 0.05; Figure 3c) and preproET-1 mRNA (three-way ANOVA, *Treatment *main effect, p = 0.04; Figure 3d) in WT and TNF mice. The greater responses in the lungs of TNF mice (*Treatment *× *Genotype *interaction, p > 0.05) is consistent with a higher particulate dose (64 ng/cm^2^) relative to WT mice (32 ng/cm^2^). After accounting for parenchymal surface area, the relative effect of PM per unit dose in WT and TNF animals was similar, despite the wide difference in basal steady-state expression levels.

### Microarray processing and quality control

**Figure 4:**
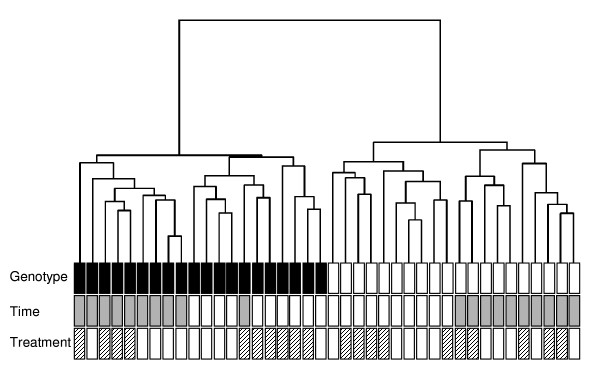
**Hierarchical clustering of arrays to examine structure at the sample level**. All 40 arrays were clustered by average linkage using block-adjusted data without prior gene selection. The bars at the base of the figure display the factors *Genotype *(WT, white; TNF, black), *Time *(0 h, white; 24 h, grey), and *Treatment *(air, white; PM, hatched) for each sample.

Agilent 22K oligonucleotide arrays were used to assess pulmonary gene expression in the experimental animals (1 lung sample/array, n = 5 arrays/treatment, 40 arrays total). Normalization of arrays resulted in a median ratio of 1 and an equivalent spread of data for all arrays [see Additional file 1]. The average background signal was 202 ± 35 for the Cy5 (sample) channel and 341 ± 101 for the Cy3 (reference) channel. Of the 20968 probes, approximately 61% of sample channel spots were identified as having a signal above background (spots flagged: 8213 ± 838 for the sample channel; 11649 ± 706 for the reference channel; 12303 ± 673 for both channels). Hierarchical clustering of arrays was carried out on lowess normalized data to identify structure at the sample level [37] (Figure 4). The main branch of the tree divided the arrays into two groups determined by factor *Genotype*. After clustering according to *Genotype*, most samples clustered by factor *Time*, although the distance between successive clusters was relatively small. In contrast to the fairly tight clustering with respect to factors *Genotype *and *Time*, there was little apparent clustering on factor *Treatment *PM. As no gene selection criteria were used to generate this visualization, the results indicate that *Genotype *effects are highly reproducible, and also provide some evidence of effects related to duration of recovery post-exposure.

### MAANOVA

**Figure 5:**
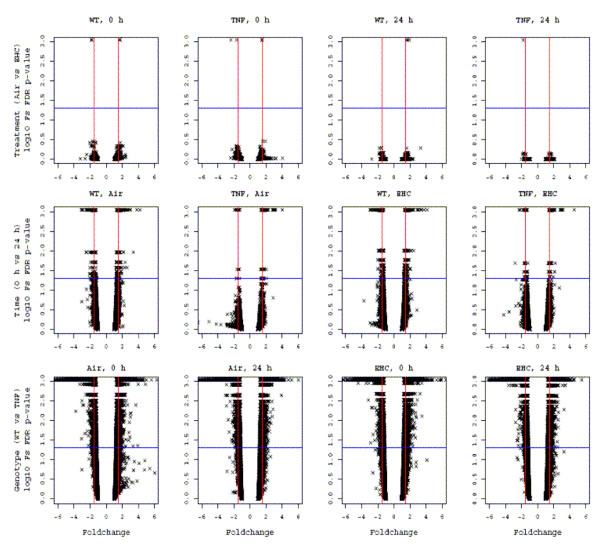
**Volcano plots of array data**. Microarray data analysed in MAANOVA by Fs FDR-adjusted p-value for each pair-wise comparison (y-axis) is represented relative to fold-change (x-axis) within factors *Treatment*, *Time*, and *Genotype*. Probes are represented by "x". The horizontal line represents a false discovery rate (FDR)-adjusted p value of 0.05. Vertical lines represent a fold-change of 1.5-fold. Note that results displayed here represent post-hoc analysis and do not respect the hierarchy of testing conducted.

To verify that the substantial differences in pulmonary gene expression between WT and TNF mice did not violate the common variance assumption, we tested the underlying empirical distribution of the residuals for *Genotype *using the Kolmogorov-Smirnov test. For all but one probe (20867 of 20868), there was insufficient evidence to conclude that the common-variance assumption was violated. Differential expression according to the factors *Treatment*, *Time*, and *Genotype *was assessed by MAANOVA, with FDR-adjusted p < 0.05 considered significant. A visual representation of fold-change and statistical significance for each pairwise comparison revealed substantial differential expression attributable to factor *Genotype*, some differential expression attributable to factor *Time*, and little differential expression attributable to factor *Treatment *(Figure 5), consistent with the array clustering. Upper and lower bounds for the number of probes significantly affected (FDR-adjusted p < 0.05) were defined according to the intersection and union of probes identified in all pairwise comparisons involving the factor (i.e. *Treatment*, *Time*, or *Genotype*) in question (Table 1). TNF mice had significantly altered expression of 1864–3331 probes (intersection-union) compared to WT mice. There was a lesser effect for factor *Time*, with 21–291 probes found to be significantly different between the two time-points. Eight probes were differentially expressed in any *Treatment *comparison (*Treatment *× *Genotype *× *Time *interaction, FDR-adjusted p < 0.05), and of these, only four were deemed present (AK020160, RIKEN cDNA 6720475J19 gene; XM_138945, RIKEN cDNA 4921531P07 gene; XM_149258, zinc finger protein 217; NM_007657, CD9 antigen). None were common to more than one pairwise comparison group. Complete lists of differentially expressed genes are available in the NCBI Gene Expression Omnibus (Accession # GSE11037; ).

**Table 1:** Number of differentially expressed probes for all pair-wise analyses as determined by MAANOVA or fold-change cut-off.

Pairwise Comparison		Significant probes (# above background)^a^	Probes with > 1.5 FC^b^
Treatment	WT,0 h (Air vs. EHC)	4 (2)	147
	TNF, 0 h (Air vs. EHC)	2 (2)	211
	WT, 24 h (Air vs. EHC)	1 (0)	147
	TNF, 24 h (Air vs. EHC)	1 (0)	117
	
Time	WT, Air (0 vs. 24 h)	279 (277)	440
	TNF, Air (0 vs. 24 h)	34 (34)	269
	WT, EHC (0 vs. 24 h)	177 (175)	486
	TNF, EHC (0 vs. 24 h)	57 (56)	349
	
Genotype	0 h, Air (WT vs. TNF)	3303 (3208)	2424
	24 h, Air (WT vs. TNF)	2332 (2328)	2279
	0 h, EHC (WT vs. TNF)	2447 (2438)	2557
	24 h, EHC (WT vs. TNF)	2285 (2282)	2232

Summary^c^			

Treatment	Union	8 (4)	572
	Intersection	0 (0)	0

Time	Union	291 (286)	1055
	Intersection	21 (21)	46

Genotype	Union	3331 (3238)	3024
	Intersection	1864 (1863)	1327

^a^Number of statistically significant probes for each comparison after analysis of three-way and two-way interactions and all main effects. Numbers in parentheses indicate probes that have been declared present in at least 4 out of 5 arrays in the group showing a significant effect.

^b^List of all probes with fold-change (FC) > 1.5; no statistical filtering.

^c^Summary of pair-wise comparisons. Union refers to the number of probes present in any comparison for a given factor. Intersection refers to the number of probes commonly affected in all comparisons for a given factor.

### Functional analysis of Genotype effects

To determine the biological significance of genotype-related differences, functional analysis was performed on the list of genes common to all genotype comparisons (1864 genes). The list of enriched terms was dominated by terms related to immune and inflammatory responses, consistent with the lung phenotype (Table 2). As expected, TNF-α was among the most highly differentially expressed genes in TNF mice compared to WT animals (18-fold increase), as was the acute phase protein serum amyloid A3 (31-fold increase), C-type lectin domain family 4 member d (19-fold increase), and a number of chemokine and immunoglobulin genes. In contrast, genes with the lowest expression levels compared to WT included the collagen binding protein procollagen c-terminal proteinase enhancer protein 2 (6-fold decrease), CYP1A1 (6-fold decrease), and genes involved in cytoskeleton organization and biogenesis.

**Table 2:** Functional analysis of probes that differ according to factor *Genotype*.*

Functional term	Number of genes	% of total	P-value
immune system process	167	9.12%	4.19 × 10^-27^
antigen processing and presentation	34	1.86%	1.61 × 10^-15^
cell activation	56	3.06%	4.48 × 10^-9^
response to wounding	66	3.60%	4.55 × 10^-9^
leukocyte activation	53	2.89%	6.90 × 10^-9^
defence response	81	4.42%	3.19 × 10^-9^
response to external stimulus	87	4.75%	3.48 × 10^-8^
cytoskeleton organization and biogenesis	87	4.75%	6.72 × 10^-8^
hemopoietic or lymphoid organ development	54	2.85%	5.14 × 10^-7^
inflammatory response	47	2.57%	1.28 × 10^-6^
taxis	29	1.58%	4.19 × 10^-6^
mast cell activation	9	0.49%	1.45 × 10^-5^
lysosome organization and biogenesis	9	0.49%	2.83 × 10^-5^
cell division	44	2.40%	8.12 × 10^-5^

* DAVID functional annotation analysis  was carried out using the list of the genes responding according to factor *Genotype *common to all treatment groups (1864 probes; 1831 unique DAVID IDs), with the Agilent Mouse Microarray G4121A probe list serving as background. Representative enriched terms selected from the top 50 of 278 gene ontology terms with a modified Fisher's Exact p < 0.05 are displayed.

### Functional analysis of Time effects

Functional analysis of differentially expressed genes according to factor *Time *revealed enrichment of apoptosis and immune/defence/stress response terms (Table 3). Enriched terms were consistent across treatment within each genotype (data not shown), indicating that any PM effects did not substantially alter effects related to duration of recovery post-exposure. Among the genes with greatest fold-changes were angiopoeitin-like 4 (4-fold), the mitogen activated protein kinase Map3k6 (3-fold), metallothionein-II (2-fold), and other genes known to respond to stress and circulating glucocorticoids. Genes with lower expression immediately after exposure compared to 24 h later included lymphotoxin B (3-fold in WT, no response in TNF animals) and a number of genes involved in immune processes.

**Table 3:** Functional analysis of probes that differ according to factor *Time*.*

Functional term	Number of genes	% of total	P-value
response to biotic stimulus	29	10.28%	3.86 × 10^-6^
defence response	28	9.93%	5.78 × 10^-6^
immune response	24	8.51%	2.23 × 10^-5^
apoptosis	19	6.74%	5.59 × 10^-4^
intracellular signalling cascade	28	9.93%	5.94 × 10^-4^
lipid catabolism	7	2.48%	0.001
protein amino acid methylation	4	1.42%	0.004
nitrogen compound catabolism	5	1.77%	0.009
response to stress	22	7.80%	0.009
angiogenesis	7	2.48%	0.01

* DAVID functional annotation analysis  was carried out using the list of all genes responding according to factor *Time *(286 probes; 282 unique DAVID IDs), with the Agilent Mouse Microarray G4121A probe list serving as background. Representative terms selected from 50 gene ontology terms with a modified Fisher's Exact p < 0.05 are displayed.

### PCR validation of microarray data

**Figure 6:**
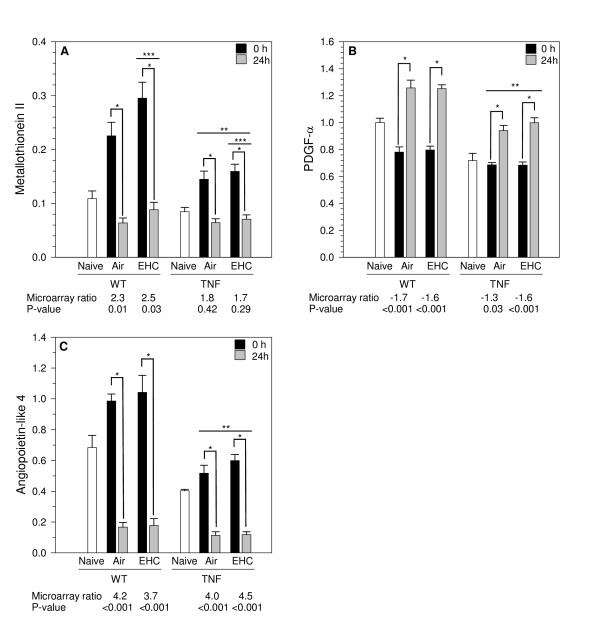
**PCR validation of *Time *effects**. Real-time PCR was used to measure expression of select probes chosen from the list of differentially expressed genes according to factor *Time *(FDR adjP < 0.05). Values represent geometric mean ± SD (n = 5 animals/group). Units are arbitrary. Measurements for naïve animals (n = 4/genotype) are included to facilitate interpretation of effects. The microarray data (the ratio of 0 h/24 h expression and the associated FDR-adjusted p-value) are displayed below each graph for comparison. A) Metallothionein-II. *Time *main effect, p < 0.001; *Genotype *main effect, p = 0.003; *Treatment *main effect, p = 0.03. B) Platelet-derived growth factor (PDGF)-α. *Time *main effect, p < 0.001. *Genotype *main effect, p < 0.001. C) Angiopoietin-like 4. *Time *main effect, p < 0.001; *Genotype *main effect, p < 0.001. Asterisks denote statistical significance (Holm-Sidak, p < 0.05). * 0 vs. 24 h within *Time*; ** WT vs. TNF within *Genotype*; *** Air vs. EHC within *Treatment*.

To validate microarray results, real-time PCR was performed on selected transcripts identified as differentially expressed according to factor *Time*. Expression was compared to naïve (unexposed) animals to facilitate interpretation of *Time *effects (Figure 6). Effects identified by microarray analysis were consistently validated by real-time PCR. A stress-responsive gene, metallothionein-II (A_51_P246317) exhibited higher expression in experimental animals of both genotypes immediately after exposure to clean air or PM, with expression returning to naïve levels 24 h later (*Time *main effect, p < 0.001; Figure 6a). Metallothionein-II levels were lower in TNF animals compared to WT animals (*Genotype *main effect, p = 0.003). In contrast, expression of platelet-derived growth factor (PDGF)-α (A_51_370090) was decreased modestly immediately after exposure, but increased 24 h later in both genotypes (*Time *main effect, p < 0.001; Figure 6b). PDGF-α mRNA levels were lower in TNF mice than in WT animals (*Genotype *main effect, p < 0.001). Expression of angiopoeitin-like 4 (A_51_P338443) was lower in both genotypes 24 h post-exposure (*Time *main effect, p < 0.001; Figure 6c). In all cases, the magnitude of fold-change of transcripts for the 0 vs. 24 h post-exposure comparisons corresponded well with fold-changes calculated using microarray data.

Interestingly, although metallothionein-II was selected for PCR validation based on the *Time *effect, with no significant PM effect identified by the array analysis, there was a statistically significant increase in the levels of this transcript after particle inhalation when measured by PCR (*Treatment *main effect, p = 0.03; Figure 6a). In contrast, of the 4 probes identified by MAANOVA as exhibiting PM-dependent changes, real-time PCR failed to confirm significant differential expression in the three probes for which primers were available (RIKEN cDNA 4921531P07 gene, zinc finger protein 217, CD9 antigen), suggesting that these results were false-positives (data not shown).

### Gene set analysis

Gene set analysis of probes selected with relaxed statistical stringency may detect subtle effects not identified by stringent statistical analysis, and so this approach was used to search for PM effects. Screening for gene ontology term enrichment in gene lists of the top 50 genes according to unadjusted p-value for all pairwise comparisons failed to identify coherent functional grouping of differentially expressed genes for factor *Treatment *[see Additional file 2]. In contrast, functional analysis of *Time *effects revealed enrichment of terms such as immune response, apoptosis, signalling pathways, blood vessel development and lipid metabolism [see Additional file 3], consistent with the analysis using all probes with FDR-adjusted P < 0.05 (Table 3), indicating that the lists were sufficiently large and representative of effects to enable identification of biological processes.

### Fold change (FC)

A fold-change cut-off approach can be used as a non-statistical method to determine a list of candidate genes, with the most likely candidates being probes appearing in multiple lists across the treatment groups. Probe lists generated by filtering on FC = 1.5 resulted in the selection of 1327–3024 (*Genotype*), 46–1055 (*Time*), and 0–572 (*Treatment*) probes (intersection-union; Table 1). Again, no probes were common to all air vs. PM comparisons. However, two probes were increased by at least 1.5-fold immediately after PM exposure in both WT and TNF mice, and were therefore chosen as top candidates for PCR validation: A_51_P279693 (NM_009992; CYP1A1; FC = 1.5 in WT, 1.9 in TNF; previously validated by real-time PCR) and A_51_P442894 (AK048310; RIKEN cDNA 1700055N04 gene, aldehyde dehydrogenase 3 family, member B2; Aldh3b2; FC = 1.5 in WT, 1.6 in TNF). While altered CYP1A1 expression was confirmed (Figure 3c), real-time PCR analysis indicated that the *Treatment *effect on Aldh3b2 mRNA was not significant (data not shown).

### One-colour analysis

A reference design was used in the present study for its simplicity, flexibility, and ease of data analysis for multi-factorial experiments [38]. It is, however, well-recognized that a reference design may add variability as a result of dividing by a potentially uncertain measurement, which could reduce the power to detect treatment effects or contribute to the generation of spurious results. Focussing on PM effects, a one-colour analysis was conducted within each genotype to verify that use of a common reference did not impede detection of effects. Only three unidentified proteins in WT mice (NM_025901, AK029835, AK036646), and one probe in TNF mice (D83146, *Mus musculus *mRNA for Six5_ partial cds; FC = 1.25) were differentially expressed according to *Treatment*. None of these genes were common to both one and two-colour analyses. The lack of PM effects in the one-colour analysis is in line with the two-colour analysis and indicates that removal of the common reference did not improve detection of statistically significant PM effects.

### Power calculations

**Figure 7:**
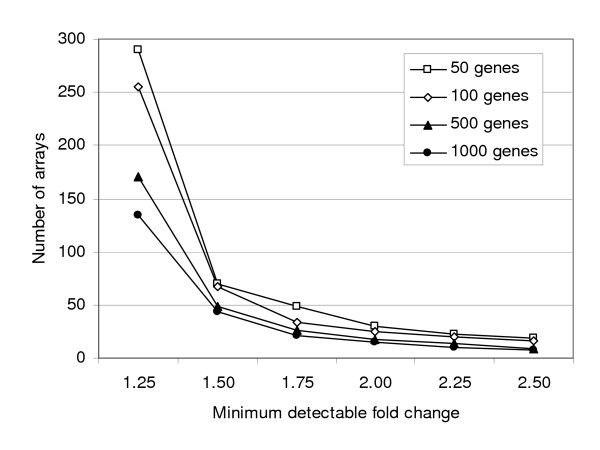
**Power simulation using data from the present microarray study**. The sample size assessment was conducted to determine the number of arrays required to detect a given fold change difference between exposed and control samples. Testing for each genotype and time point group resulted in four independent assessments for each fold-change analysis. Results were then averaged, and the number of arrays required to detect a given fold-change was plotted relative to the minimal detectable fold change. From upper to lower, the lines represent simulations in which 50, 100, 500, and 1000 genes were set as differentially expressed at the fold-change indicated on the x-axis.

Because of the lack of measurable PM effect on pulmonary gene expression, we carried out power simulations on two-colour data to determine the sample size required to detect differential gene expression due to *Treatment *using array data from the present study. Data generated for all four analyses (WT, 0 h; WT, 24 h, TNF, 0 h, TNF, 24 h) were used in the power calculation and averaged (Figure 7). As expected, the power simulations show an inverse relationship between the number and magnitude of changes expected and the number of arrays required. The data indicate that with the small effect size in this study, a large number of biological replicates would be required (e.g. at least 20 per group for a fold-change < 1.8).

## Discussion

Despite evidence of transcription factor activation in lung cells after PM exposure [16], few studies have examined effects of PM on pulmonary gene expression (reviewed in [17]). We hypothesized that by evaluating differential gene expression at the global transcript level, a microarray approach would enable identification of PM-activated pathways not investigated by conventional approaches. In the present work, the impact of PM inhalation on transcript profiles in healthy mice and in mice with chronic lung inflammation was assessed immediately and 24 h post-exposure to detect effects that could be related to the rapid response indicated by epidemiologic studies [26,27]. Using real-time PCR, we confirmed positive effects of PM inhalation on three distinct pathways: 1) up-regulation of CYP1A1, relevant to the presence of combustion by-products in the inhaled urban PM and activation of a procarcinogenic pathway; 2) up-regulation of metallothionein-II, relevant to the presence of toxic metals in the inhaled particles and activation of pathways to protect against metal toxicity and oxidative stress; and 3) up-regulation of ET-1, relevant to endothelial dysfunction generated by deposited toxicants and downstream cardiovascular effects. The activation of CYP1A1 and metallothionein-II is in line with our previous report relating chemical composition with the biological potency of particles [34]. These responses establish delivery of a biologically-effective internal dose in our study despite the absence of visible impacts on lung lavage cytology, and are consistent with previous work showing that pronounced inflammation and acute lung injury are not prerequisites for induction of physiologically-relevant pathways by PM [9,14]. The early time points assessed reduce the likelihood that interpretation of molecular data would be confounded by infiltrating inflammatory cells, which often peak 24–48 h after acute injury. Remarkably, although microarray analysis identified significant differential gene expression attributable to genotype and to duration of recovery post-exposure, no significant PM effects were found. Since array quality was internally validated by detection of positive effects, the data indicate that any effects of inhaled particles were below the threshold of detection.

As the site of gas exchange, the lungs are continuously exposed to airborne contaminants, and possess innate defence mechanisms that facilitate clearance of inhaled particles without provoking excessive inflammation that could lead to tissue damage. Chronic inflammation can impair lung defences by increasing the permeability of the epithelial barrier and slowing mucocilliary clearance, facilitating passage of inhaled particles into the tissue [10]. Depletion of anti-oxidant defences, activation of redox-sensitive transcription factors, and chromatin remodelling in the inflamed lungs could all alter the transcriptional response to PM [13]. Microarray data confirmed that the pulmonary transcriptome of TNF mice was substantially different from that of WT littermates, with activation of inflammatory and immune response pathways consistent with the lung phenotype [29,30]. Clearly, differential expression detected in this model reflects import of message due to changes in cellularity, such as influx of inflammatory cells, in addition to strict transcriptional activation by TNF-α. Surprisingly, this persistent inflammatory state did not measurably amplify or alter the response to inhaled particles. It is unclear whether the lack of response is due to functional transcription-independent defences in TNF animals, or to insufficient sensitivity to detect subtle PM effects by microarray at the whole lung level. Despite the higher numbers of inflammatory cells in the lungs of TNF mice, levels of ρ-tyrosine, a product of hydroxylation of phenylalanine indicative of oxidative stress, are not significantly higher compared to WT littermates, nor increased in either genotype after repeated exposure to PM and ozone [33]. This suggests that anti-oxidant defences may still be functional in the TNF model. After adjusting for the decreased alveolar surface area in TNF mice [30], PM effects on CYP1A1 and ET-1 were remarkably similar to those observed in WT animals. These results indicate that the altered expression of specific genes relevant to adverse PM effects was robust to the physiological status of the lungs, despite significant genotype differences in the basal levels of the transcripts. The lower basal metallothionein-II mRNA level and apparent reduced response in TNF mice could reduce scavenging of toxicologically-relevant metals [34,39].

The data indicate that effects of urban particles on the lung transcriptome following a single acute exposure by inhalation are subtle. The EHC-6802 urban particle preparation used in this study is an environmental material collected in Ottawa, Canada, and is equivalent to the well-characterized EHC-93 particle preparation [9,18,34]. The inherent toxicity of EHC-93 particles has been demonstrated *in vivo *using intratracheal instillation [39,40], and inhalation studies have revealed endothelin system activation in the absence of pronounced inflammatory changes [14,18,19]. Activation of biologically-relevant pathways by EHC-6802 is confirmed here by real-time PCR. Thus, the lack of overt effects detected by microarray analysis is not likely due to an absence of factors associated with health effects of particles in this particle preparation. It remains possible that repeated exposure to elevated PM levels could induce measurable effects that were not apparent after the single exposure used here. In the only other microarray study published in the literature that has, to our knowledge, examined pulmonary effects of urban PM in an inhalation model, subchronic exposure to concentrated ambient PM yielded little evidence of differential expression in the whole lung [24]. Note that in this previous study expression was measured 3–4 days after the last exposure, and may have missed subsiding transcriptional events. In the present study, tissues were recovered immediately and 24 h post-exposure to capture rapid effects of exposure on gene expression. Statistical analysis of array data was conducted at varying levels of stringency such that the absence of detected effects was unlikely to be due to overly stringent filters. Moreover, sample and array quality were confirmed by detection of *Time *and *Genotype *effects in line with expected responses. Compared with the coherence and PCR validation of *Time *and *Genotype *effects, the lack of consistent *Treatment *effects suggests that the few probes identified as responsive to PM were false-positives, and that the absence of PM effects was not due to the analytical approach chosen. Rather, the data suggest that overt, coordinated transcriptional responses, such as would be expected in an inflammatory signalling cascade, may not be a major part of the pulmonary response to inhaled urban particles in this model.

Internal dose and method of delivery should be considered when comparing results across studies. Previous micro- and macroarray studies examining PM effects on pulmonary gene expression include exposure to saline suspension of urban PM [23,25], concentrated ambient particles [24], diesel exhaust particles [41], residual oil fly ash [42], and ultrafine carbon particles [43]. Studies using an intratracheal instillation approach employed particle doses ranging from 1.25–10 mg, delivered as a bolus in 0.5 mL saline [23,25,41,42]. In the present study, the dose of EHC-6802 within the respiratory compartment was estimated to be 16 μg (32 ng/cm^2 ^for WT mice and 64 ng/cm^2 ^for TNF mice). The estimated doses were only 25-fold (WT mice) or 50-fold (TNF mice) higher than the modelled deposition for a plausible 24 h human exposure scenario (1.3 ng/cm^2 ^[28]), but more importantly, were 100–1000 times lower than the dose of particles delivered in the intratracheal instillation studies cited above. Exposure by inhalation, a lower internal dose of PM, and a slower dose rate are less likely to provoke an inflammatory response that could impact transcript levels, and indeed did not cause a significant influx of inflammatory cells, in agreement with our previous work in rats [9,14]. Our inhalation approach should therefore be more sensitive to changes in parenchymal gene expression and avoid artifacts of an overly high internal dose of particles. For ethical reasons, nose-only exposures should be kept to a minimum duration, and therefore the dose-rate in our study was considerably higher than for a 24 h environmental exposure. Nevertheless, the pulmonary deposition of PM in our inhalation study is directly relevant to the human experience, once a number of reasonable uncertainty factors are considered. These include possible decreased potency of EHC-6802 by comparison to fresh particles, interspecies differences in sensitivity to air pollutants, and the heightened sensitivity within a subset of the human population, such as the known increased adverse risk of individuals with COPD, congestive heart failure, and atherosclerosis [4,5].

While training in nose-only exposure tubes in the days preceding exposure is aimed at reducing animal stress, exposure altered the expression of a number of genes in the lungs, even in animals exposed to filtered air alone. These changes show some similarity with effects of stress on other organs, such as the spleen [44], and likely relate to systemic effects of stress. Since both air and PM-exposed animals were similarly exposed to the inhalation system in the present study, such changes do not confound interpretation of results. Moreover, within each genotype, air and particle exposed animals exhibited similar *Time *effects. It is, however, not known to what degree these changes may impede detection of PM effects in the subset of genes affected. It is noteworthy that although metallothionein-II was selected for PCR validation based on its response to *Time*, with no significant PM effect according to array analysis, expression was significantly increased by PM when measured by the more sensitive real-time PCR method. The higher levels of metallothionein-II mRNA in PM-exposed animals indicate that, at least for this gene, effects of exposure to PM and stress were additive, consistent with work showing that the mechanism of metallothionein-II induction by restraint stress is distinct from that by chemical stress [45].

Toxicogenomic analysis *in vivo *at doses relevant to human health can be problematic when subtle changes are expected to arise. The present study shows that inhaled particles at an internal dose relevant to the human experience may not cause significant changes in gene expression detectable at the level of the whole lung using a moderate number of microarrays. Moreover, small but physiologically-relevant changes in gene expression, such as activation of endothelin and xenobiotic-metabolism pathways, may not be identified by microarray analysis. To detect statistically significant differential expression of a few probes from among 21,000 requires relatively large effect sizes, or a very high number of arrays. Using our data and taking a conservative approach of 90% confidence, we calculated that 60–70 arrays would be required to detect 1.5-fold PM effects in animals of a given genotype, assuming 100 differentially expressed genes with FDR-adjusted p < 0.05 (Figure 7). Experiments requiring this number of arrays may not be practical for many laboratories. Using fewer arrays and an arbitrary fold cut-off rather than statistical analysis risks inclusion of false-positive results and/or exclusion of small (< 1.5-fold) but physiologically relevant changes. This type of approach would require extensive RT-PCR on multiple biological replicates to validate any findings. Alternatively, taking a more traditional hypothesis-driven approach through selection of probe subsets diminishes the likelihood of discovering unexpected effects. A third possibility, increasing dose to generate larger effect sizes, may create artifacts such as overload of clearance pathways. In light of our observations, there are several approaches that might be considered for the assessment of pulmonary effects of contaminant exposure. Since experiments examining expression in whole lung tissue may be insensitive to focal responses in target cells, an anatomically-biased or cell-type specific approach such as laser-capture microdissection might uncover local impacts of PM inhalation using fewer arrays. This should be fairly straightforward for the study of effects in WT animals. However, complex structural changes and intensive inflammatory infiltration will complicate analysis in models such as the TNF mice. Secondly, appropriate pooling of animals may reduce the impact of biological variability, provided a sufficient number of arrays hybridized with independently-pooled RNA are used to permit statistical analysis [46,47]. However, it is likely that a large number of arrays would still be required to detect subtle effects. Lastly, a combination of transcriptome screening and conventional toxicologic analyses may be appropriate to examine subtle effects of exposure.

## Conclusion

The present study verified increased expression of ET-1, CYP1A1, and metallothionein-II in both wildtype and TNF mice exposed by the nose-only inhalation route, consistent with *in vitro *[34] and *in vivo *[19,35] analyses, and in line with our understanding of the impact of PM on cardiovascular, toxicant metabolism, and metal-responsive pathways. These effects were robust to the physiological status of the lungs, and remarkably, did not differ greatly between genotypes. Although the particles clearly induce pathways relevant to endothelial dysfunction (ET-1), carcinogenicity (CYP1A1), and metal toxicity (metallothionein-II), the exposures did not measurably initiate inflammation (wildtype) or exacerbate existing inflammation (TNF) in the lungs as measured by lavage cytology and global pulmonary gene expression. Given the increased susceptibility of individuals with respiratory disease [4-7], it may be that susceptibility arises as a result of the interaction of primary effects with host factors, in addition to any enhancement or alteration of these primary effects. For example, individuals with existing endothelial dysfunction and an ineffective compensation for the vasopressor effect of ET-1 may respond adversely to a PM-induced increase of circulating ET-1, while healthy individuals may compensate with release of nitric oxide and prostacyclin and not exhibit hemodynamic changes in response to the same relative increase of circulating ET-1. While microarray analysis is a valuable approach for genome-wide screening of pronounced effects, such as characterization of transcriptional changes resulting from TNF over-expression, it may not be particularly useful for dissecting pathways at the level of the whole lung after delivery of an environmentally-relevant internal dose of contaminants. Increased power, site-specific analysis, or more sensitive animal models may be required to investigate effects of acute particle exposure on pulmonary gene expression. In addition, focussed approaches targeting key endpoints should be considered to complement genome-wide transcript profiling. Our data support the hypothesis that adverse health effects of acute exposure to urban PM may be dominated by physiological response cascades rather than widespread changes in the expression of genes escaping homeostatic controls. Nevertheless, transcriptional activation of certain key pathways, such as endothelin synthesis and xenobiotic transformation, may be relevant to these health effects.

## Methods

### Animals

SP-C/TNF-α mice were provided by Dr. R. J. Mason (National Jewish Medical and Research Centre, Denver, CO, USA) and crossed with C57BL/6 mice (Charles River Laboratories, St. Constant, QC, Canada). Note that the SP-C/TNF-α line has been maintained as a heterozygous line by repeated backcrossing to C57BL/6 mice for over a decade [29,30,33]. Male transgenic TNF mice and their WT littermates were genotyped by PCR analysis of genomic DNA [29]. Animals were exposed as three cohorts, each approximately 3 months apart, and were 131 ± 5 days old and 28.9 ± 2.8 g (WT: 30.8 ± 2.5 g; TNF: 27.1 ± 1.7) at the time of exposure. Mice were housed in individual plexiglass cages on wood-chip bedding under HEPA-filtered air and held to a 12 h dark/light cycle. Food and water were provided *ad libitum*. All experimental protocols were reviewed and approved by the Animal Care Committee of Health Canada as set forth in the Guidelines of the Canadian Council on Animal Care.

### PM preparation

The urban particles EHC-6802 consist of a blend of total suspended PM recovered from filters of the single-pass air-purification system at the Environmental Health Centre (Tunney's Pasture, Ottawa, ON, Canada) in 1996, 1998, 2000, and 2002. Particles were mechanically sieved using a 36 μm mesh filter, and combined in equal proportions. EHC-6802 was recovered at the same site and in the same manner as the urban particles EHC-93 [9,18,34], and chemical characterization of the EHC-6802 material has confirmed that it is equivalent to the EHC-93 material (data not shown).

### Activation of the aryl hydrocarbon receptor (AhR) by EHC-6802

The AhR-based luciferase reporter cell line H1L1.1c2 (kindly provided by Dr. M. S. Denison, University of California, Davis, CA, USA) was used to assess AhR-activation by the EHC-6802 particles. H1L1.1c2 cells were exposed in triplicate to EHC-6802 (1, 10, 100 μg/cm^2^), 10 nM benzo(a)pyrene (positive control), and TiO_2 _(negative control) in 24-well plates (2 cm^2^/well) for 4 h, and luciferase activity was measured as previously described [48].

### Inhalation exposure

Mice were trained in nose-only exposure tubes over 4 consecutive days and then exposed for 4 h to clean air or 42 mg/m^3 ^EHC-6802 by nose-only exposure as described previously [9,19]. Particle concentration was monitored during each exposure at the inhalation ports by isokinetic sampling using 0.2 μm Teflon filters. Filter weight was divided by the sampling volume to provide a direct estimate of the time-weighted average particle concentration. Particle counts and size measurements were performed at the inhalation ports (optical size range of 0.3–10 μm; Lasair Model 301; Particle Measuring Systems, Boulder, CO, USA). Aerodynamic size characteristics were determined by gravimetric cascade impactor analysis of isokinetic samples at the inhalation ports (seven-stage Mercer cascade impactor, 1 L/min, 0.2–5.1 μm effective cut-off diameter; Intox, Albuquerque, NM, USA) or the chamber exhaust (seven-stage Mercer cascade impactor, 10 L/min, 0.2 to 9.8 μm effective cut-off diameter; Intox). Multimodal particle size distribution analysis [49] and pulmonary deposition modelling were performed as previously described [18]. Deposition model assumptions for mice were strict nasal breathing, a minute ventilation of 34 mL/min, an alveolar surface area of 500 cm^2^, and a tracheobronchial surface area of 3.5 cm^2 ^(default values for the Regional Deposited Dose Ratio RDDR2 modelling software, US EPA).

### Collection of biological samples

Mice were anaesthetized by administration of sodium pentobarbital (60 mg/kg, i.p.) and euthanized by exsanguination immediately or 24 h after termination of exposure (n = 5 animals of each genotype/treatment/time). Lungs were washed by bronchoalveolar lavage with warm saline (37°C) at 30 mL/kg body weight, then flash frozen in liquid nitrogen and stored at -80°C until isolation of RNA. Cells recovered by centrifugation (1500 rpm for 10 min at 4°C) were counted in a Coulter Multisizer II (Coulter Canada, Ville St. Laurent, QC, Canada) and differential cell counts were obtained from cytospin preparations. Lungs were homogenized in TRIzol and RNA was isolated according to manufacturer's instructions (Invitrogen Canada Inc., Burlington, ON, Canada), and further purified using RNeasy Mini Kits (Qiagen Inc., Mississauga, ON, Canada). RNA was quantified using the RiboGreen RNA Quantitation Reagent and Kit (Molecular Probes, Eugene, OR, USA), and quality was confirmed using the Agilent 2100 Bioanalyzer and RNA 6000 NanoLab Chip Kit (Agilent Technologies Canada Inc., Mississauga, ON, Canada). For histology, lungs of unexposed animals (n = 3/genotype) were inflated in chest at 25 cm H_2_0 static pressure by intratracheal instillation of 4% paraformaldehyde in phosphate-buffered saline. The lungs were excised, immersed in fixative, and stored at 4°C. Tissue blocks were dehydrated in ethanol and embedded in paraffin. Sections (0.75 μm) were stained with hematoxylin and eosin.

### Real-time reverse transcription-polymerase chain reaction (RT-PCR)

Total RNA was reverse transcribed using MuLV reverse transcriptase and random hexamers according to manufacturer's instructions (Applied Biosystems, Mississauga, ON, Canada). Twenty ng of cDNA was combined with Quantitect primer assays and real-time PCR supermix (Qiagen) and run according to the product protocol on the iCycler thermal cycler (Bio-Rad Laboratories Canada Ltd., Mississauga, ON, Canada) using 50 μL volumes [50]. Post-run melt curves were routinely inspected to verify product purity. Expression was calculated relative to reference genes (β-actin and TATA binding protein) using the comparative threshold cycle method [51]. Statistical significance was assessed by three-way ANOVA with *Treatment *(Air, EHC), *Genotype *(WT, TNF), and *Time *(0, 24 h) as factors, followed by the Holm-Sidak multiple comparison procedure to elucidate the pattern of significant effects (α = 0.05; Sigma Stat 3.0, SPSS Inc., Chicago, IL, USA). PM effects were represented graphically as fold-change relative to the time and genotype-matched air-exposed controls.

### Microarray hybridization

An unbalanced block factorial design [52] was used for the factors *Treatment *(0, 50 mg/m^3^), *Time *(0, 24 h), and *Genotype *(WT, TNF) and blocked for the nuisance factors *Date of exposure *and *Day of hybridization *[53]. Five biological replicates per condition were used for a total of 40 microarrays. Individual 2.5 μg aliquots of RNA from each sample were amplified and labelled using the Low RNA Input Fluorescent Linear Amplification Kit (Agilent). Agilent Mouse G4121A Microarrays (containing approximately 21,000 probes) were hybridized with 5 μg Cy5-labelled lung RNA and 5 μg Cy3-labelled Universal Mouse Reference RNA (Stratagene, CA, USA), used as a common reference on all arrays [38]. Arrays were incubated overnight at 60°C in Agilent hybridization solution and washed according to manufacturer's instructions. Arrays were scanned using a ScanArray Express (Perkin-Elmer Life Sciences, Woodbridge, ON, Canada), and data were acquired with ImaGene 5.5 (BioDiscovery, CA, USA).

### Statistical analysis of microarray data

The background signal for each array was determined using the negative control (-)3xSLv1 probe. Spots with median signal intensities within three standard deviations of the (-)3xSLv1 probe mean were flagged as absent. Lowess normalization [54] was performed using the SAS/STAT software, Version 8.2 of the SAS System for Windows (1999–2001 SAS Institute Inc., Cary, NC, USA). Data displays produced in R, including ratio intensity plots for the raw and normalized data, comparison boxplots, heatmaps, dendrograms, and volcano plots, were used for the assessment of data quality and for visual comparison of the impact of each factor [55]. The logarithm base 2 relative intensities were used for subsequent analyses.

The present experiment was designed to evaluate pollutant-phenotype interactions, and as such required the analysis of multiple factors and interactions among factors. A factorial design is a suitable statistical framework to maximize resources, and permits analysis of multiple factors with the same precision as if the experiment were designed to examine one factor, assuming common variance. The Microarray Analysis of Variance (MAANOVA) library [56] in R was used to identify differentially expressed genes. The statistical model included the main effects *Treatment*, *Time*, and *Genotype*, the three-way interaction, and all two-way interactions. The Fs statistic [57], a shrinkage estimator for the gene-specific variance components, was used, and p-values for all statistical tests were estimated using the permutation method (1000 permutations with residual shuffling). P-values were then adjusted for multiple comparisons using the false discovery rate (FDR) approach [58]. Group means for the fold-change calculation were based on the adjusted relative intensity for each gene after subtracting estimated *Date of exposure *and *Day of hybridization *effects from the normalized ratio. Genes with FDR-adjusted p < 0.05 and present on at least 4 of 5 arrays in the group showing a significant effect were considered differentially expressed. Gene ontology enrichment analysis was performed using the Database for Annotation, Visualization and Integrated Discovery (DAVID) [59], available at . Agilent GeneSpring GX was used for filtering on fold-change. Microarray data were deposited in the NCBI Gene Expression Omnibus (Accession # GSE11037; ).

### Power simulations

Power scenarios were investigated using the samr.assess.samplesize function in the SAMR library [60] to determine the sample size (n animals, 1 array/animal) required to detect 1.25–2.5 fold changes in expression when comparing control and exposed animals for each genotype at each time point. The required sample size was identified through inspection of plots generated from this application, and corresponded to the value at which the 90^th ^percentile for FDR was less than 0.05. Results for the four analyses were averaged for each fold change, and the number of arrays required was plotted relative to the minimum detectable fold-change.

## Competing interests

The authors declare that they have no competing interests.

## Authors' contributions

EMT designed the study, carried out the microarray and PCR analyses, contributed to the data interpretation, and drafted the manuscript. AW contributed to the study design and performed the statistical analyses. CLY contributed to the study design and data interpretation. RV conceived of the study and contributed to its design, deployment, and interpretation as principal investigator. All authors read and approved the final manuscript.

## Supplementary Material

Additional file 1 **Box-plots of array data for all 40 arrays used in the study**. The central line represents the median of the data and the tails represent the upper (75^th^) and lower (25^th^) percentile. Click here for file

Additional file 2 **Gene ontology term enrichment for factor *Treatment*.** DAVID functional annotation analysis  was carried out using lists of the top 50 genes by unadjusted p-value according to factor *Treatment *within each genotype/time group. The Agilent Mouse Microarray G4121A probe list served as background population for the analysis. All terms within "Biological Process" with a modified Fisher's Exact p < 0.1 are listed. Click here for file

Additional file 3 **Gene ontology term enrichment for factor *Time***. DAVID functional annotation analysis  was carried out using lists of the top 50 genes by unadjusted p-value according to factor *Time *within each genotype (WT, TNF). The Agilent Mouse Microarray G4121A probe list served as background population for the analysis. All terms within "Biological Process" with a modified Fisher's Exact p < 0.1 are listed. Click here for file
