# Supplementary material for: Toxicogenomic analysis of susceptibility to inhaled urban particulate matter in mice with chronic lung inflammation
Source: Part Fibre Toxicol. 2009 Mar 11;6:6. doi: 10.1186/1743-8977-6-6 (PMC2661044; doi:10.1186/1743-8977-6-6)
Supplement: Additional file 2 — Gene ontology term enrichment for factor Treatment. DAVID functional annotation analysis was carried out using lists of the top 50 genes by unadjusted p-value according to factor Treatment within each genotype/time group. The Agilent Mouse Microarray G4121A probe list served as background population for the analysis. All terms within "Biological Process" with a modified Fisher's Exact p < 0.1 are listed. [file 1743-8977-6-6-S2.doc]

**Additional file 2.** Gene ontology term enrichment for factor *Treatment*.*

| **Term** | **Count** | **PValue** | **Genbank accession** |
| --- | --- | --- | --- |
| ***WT/0 h*** |  |  |  |
| none |  |  |  |
|  |  |  |  |
| ***WT/24 h*** |  |  |  |
| ovarian follicle development | 2 | 0.026 | NM_133893, NM_012046, |
| menstrual cycle | 2 | 0.054 | NM_133893, NM_012046, |
| female sex differentiation | 2 | 0.056 | NM_133893, NM_012046, |
| female gonad development | 2 | 0.056 | NM_133893, NM_012046, |
| development of primary female sexual characteristics | 2 | 0.056 | NM_133893, NM_012046, |
| post-embryonic development | 2 | 0.067 | NM_133893, NM_012046, |
| development of primary sexual characteristics | 2 | 0.080 | NM_133893, NM_012046, |
| gonad development | 2 | 0.080 | NM_133893, NM_012046, |
| sphingolipid metabolism | 2 | 0.085 | NM_054052, AK035314, |
| development | 9 | 0.087 | AK014697, U29148, NM_133893, NM_012046, AF323488, AK044171, NM_017392, AK020389, NM_054052, |
|  |  |  |  |
| ***TNF/0 h*** |  |  |  |
| cell-cell signaling | 4 | 0.009 | AK053672, NM_009528, NM_009793, AK052703, |
| regulation of osteoblast differentiation | 2 | 0.021 | NM_009528, NM_007855, |
| synaptic transmission | 3 | 0.027 | AK053672, NM_009793, AK052703, |
| osteoblast differentiation | 2 | 0.033 | NM_009528, NM_007855, |
| transmission of nerve impulse | 3 | 0.035 | AK053672, NM_009793, AK052703, |
| tissue development | 3 | 0.052 | NM_009505, NM_009528, NM_007855, |
| eye development (sensu Mammalia) | 2 | 0.063 | D83144, NM_009505, |
| eye development (sensu Vertebrata) | 2 | 0.066 | D83144, NM_009505, |
| negative regulation of cellular physiological process | 4 | 0.078 | D83144, BI455570, NM_009505, NM_007855, |
| organ development | 5 | 0.079 | D83144, BI455570, NM_009505, NM_009528, NM_007855, |
| negative regulation of physiological process | 4 | 0.088 | D83144, BI455570, NM_009505, NM_007855, |
|  |  |  |  |
| ***TNF/24 h*** |  |  |  |
| potassium ion transport | 3 | 0.032 | AK016808, NM_008425, AK007011, |
| biological process unknown | 6 | 0.049 | AI851834, AK030323, AK016943, AK046719, NM_019393, AK083094, |

*DAVID functional annotation analysis (<http://david.abcc.ncifcrf.gov/home.jsp>) was carried out using lists of the top 50 genes by unadjusted p-value according to factor *Treatment* within each genotype/time group. The Agilent Mouse Microarray G4121A probe list served as background population for the analysis. All terms within "Biological Process" with a modified Fisher’s Exact p<0.1 are listed.
