# Supplementary material for: Toxicogenomic analysis of susceptibility to inhaled urban particulate matter in mice with chronic lung inflammation
Source: Part Fibre Toxicol. 2009 Mar 11;6:6. doi: 10.1186/1743-8977-6-6 (PMC2661044; doi:10.1186/1743-8977-6-6)
Supplement: Additional file 3 — Gene ontology term enrichment for factor Time. DAVID functional annotation analysis was carried out using lists of the top 50 genes by unadjusted p-value according to factor Time within each genotype (WT, TNF). The Agilent Mouse Microarray G4121A probe list served as background population for the analysis. All terms within "Biological Process" with a modified Fisher's Exact p < 0.1 are listed. [file 1743-8977-6-6-S3.doc]

**Additional file 3.** Gene ontology term enrichment for factor *Time*.*

| **Term** | **Count** | **PValue** | **Genbank accession** |
| --- | --- | --- | --- |
| ***WT*** |  |  |  |
| lymphocyte activation | 5 | 0.001 | NM_011817, NM_009858, AK007630, NM_013487, Y17159, |
| immune cell activation | 5 | 0.001 | NM_011817, NM_009858, AK007630, NM_013487, Y17159, |
| cell activation | 5 | 0.001 | NM_011817, NM_009858, AK007630, NM_013487, Y17159, |
| negative regulation of enzyme activity | 3 | 0.004 | NM_011817, AK007630, NM_020581, |
| negative regulation of apoptosis | 4 | 0.004 | NM_010286, BC011432, AK007630, NM_020581, |
| negative regulation of programmed cell death | 4 | 0.004 | NM_010286, BC011432, AK007630, NM_020581, |
| intracellular signaling cascade | 8 | 0.009 | AK009928, NM_011817, NM_016693, BC011432, NM_025831, NM_133753, Y17159, NM_010407, |
| apoptosis | 6 | 0.010 | NM_011817, NM_010286, BC011432, NM_021897, AK007630, NM_020581, |
| regulation of enzyme activity | 4 | 0.011 | NM_011817, NM_016693, AK007630, NM_020581, |
| programmed cell death | 6 | 0.011 | NM_011817, NM_010286, BC011432, NM_021897, AK007630, NM_020581, |
| regulation of apoptosis | 5 | 0.011 | NM_010286, BC011432, NM_021897, AK007630, NM_020581, |
| regulation of programmed cell death | 5 | 0.012 | NM_010286, BC011432, NM_021897, AK007630, NM_020581, |
| cell death | 6 | 0.013 | NM_011817, NM_010286, BC011432, NM_021897, AK007630, NM_020581, |
| death | 6 | 0.014 | NM_011817, NM_010286, BC011432, NM_021897, AK007630, NM_020581, |
| hemopoietic or lymphoid organ development | 4 | 0.015 | NM_011817, NM_013487, NM_008518, U87620, |
| immune response | 6 | 0.018 | NM_011817, NM_009858, AK007630, NM_013487, Y17159, NM_008518, |
| regulation of protein kinase activity | 3 | 0.024 | NM_011817, NM_016693, AK007630, |
| T cell activation | 3 | 0.025 | NM_011817, NM_009858, NM_013487, |
| cell communication | 15 | 0.026 | NM_016693, NM_010728, AK009928, BC011432, NM_009858, NM_025831, NM_133753, NM_013487, Y17159, NM_020581, NM_010407, NM_011817, NM_008339, NM_008513, NM_008741, |
| regulation of kinase activity | 3 | 0.026 | NM_011817, NM_016693, AK007630, |
| regulation of transferase activity | 3 | 0.027 | NM_011817, NM_016693, AK007630, |
| development | 11 | 0.032 | AK009928, NM_011817, NM_016873, NM_008513, BC011432, NM_008969, NM_013487, NM_013594, NM_020581, NM_008518, U87620, |
| defense response | 6 | 0.036 | NM_011817, NM_009858, AK007630, NM_013487, Y17159, NM_008518, |
| positive regulation of biological process | 6 | 0.036 | NM_011817, NM_016693, BC011432, NM_021897, AK007630, NM_020581, |
| signal transduction | 14 | 0.038 | NM_016693, NM_010728, AK009928, BC011432, NM_009858, NM_025831, NM_133753, NM_013487, Y17159, NM_010407, NM_011817, NM_008339, NM_008513, NM_008741, |
| response to biotic stimulus | 6 | 0.041 | NM_011817, NM_009858, AK007630, NM_013487, Y17159, NM_008518, |
| lipid metabolism | 5 | 0.045 | NM_012006, NM_016873, BC011432, NM_008969, NM_020581, |
| phosphate metabolism | 6 | 0.053 | NM_016693, AK041572, BC017621, NM_013743, NM_013642, NM_010407, |
| phosphorus metabolism | 6 | 0.053 | NM_016693, AK041572, BC017621, NM_013743, NM_013642, NM_010407, |
| hemopoiesis | 3 | 0.079 | NM_011817, NM_013487, U87620, |
| negative regulation of protein kinase activity | 2 | 0.083 | NM_011817, AK007630, |
| negative regulation of transferase activity | 2 | 0.086 | NM_011817, AK007630, |
| phosphorylation | 5 | 0.089 | NM_016693, AK041572, BC017621, NM_013743, NM_010407, |
|  |  |  |  |
| ***TNF*** |  |  |  |
| blood vessel development | 5 | 0.002 | NM_010217, BC011432, NM_008871, NM_013655, NM_020581, |
| vasculature development | 5 | 0.002 | NM_010217, BC011432, NM_008871, NM_013655, NM_020581, |
| angiogenesis | 4 | 0.006 | NM_010217, NM_008871, NM_013655, NM_020581, |
| blood vessel morphogenesis | 4 | 0.010 | NM_010217, NM_008871, NM_013655, NM_020581, |
| lipid metabolism | 6 | 0.017 | NM_012006, BC011432, NM_007408, NM_019422, NM_020581, NM_007453, |
| enzyme linked receptor protein signaling pathway | 4 | 0.030 | NM_010217, NM_011058, AK017962, NM_011595, |
| negative regulation of apoptosis | 3 | 0.052 | NM_010286, BC011432, NM_020581, |
| protein amino acid dephosphorylation | 3 | 0.053 | AK017962, NM_008979, NM_013642, |
| negative regulation of programmed cell death | 3 | 0.054 | NM_010286, BC011432, NM_020581, |
| dephosphorylation | 3 | 0.059 | AK017962, NM_008979, NM_013642, |
| transmembrane receptor protein tyrosine kinase signaling pathway | 3 | 0.059 | NM_010217, NM_011058, NM_011595, |
| organ morphogenesis | 5 | 0.065 | NM_010217, NM_011058, NM_008871, NM_013655, NM_020581, |
| potassium ion transport | 3 | 0.068 | NM_009721, AK016808, NM_008430, |
| regulation of angiogenesis | 2 | 0.073 | NM_008871, NM_020581, |
| phosphorus metabolism | 6 | 0.078 | NM_016693, NM_011058, BC017621, AK017962, NM_008979, NM_013642, |
| phosphate metabolism | 6 | 0.078 | NM_016693, NM_011058, BC017621, AK017962, NM_008979, NM_013642, |
| response to chemical stimulus | 4 | 0.091 | NM_011338, NM_013655, AK003119, NM_007453, |

*DAVID functional annotation analysis (<http://david.abcc.ncifcrf.gov/home.jsp>) was carried out using lists of the top 50 genes by unadjusted p-value according to factor *Time* within each genotype (WT, TNF). The Agilent Mouse Microarray G4121A probe list served as background population for the analysis. All terms within "Biological Process" with a modified Fisher’s Exact p<0.1 are listed.
