# Supplementary material for: Toxicogenomic analysis of susceptibility to inhaled urban particulate matter in mice with chronic lung inflammation
Source: Part Fibre Toxicol. 2009 Mar 11;6:6. doi: 10.1186/1743-8977-6-6 (PMC2661044; doi:10.1186/1743-8977-6-6)
Supplement: Supplementary file 3 [file 1743-8977-6-6-S1.doc]

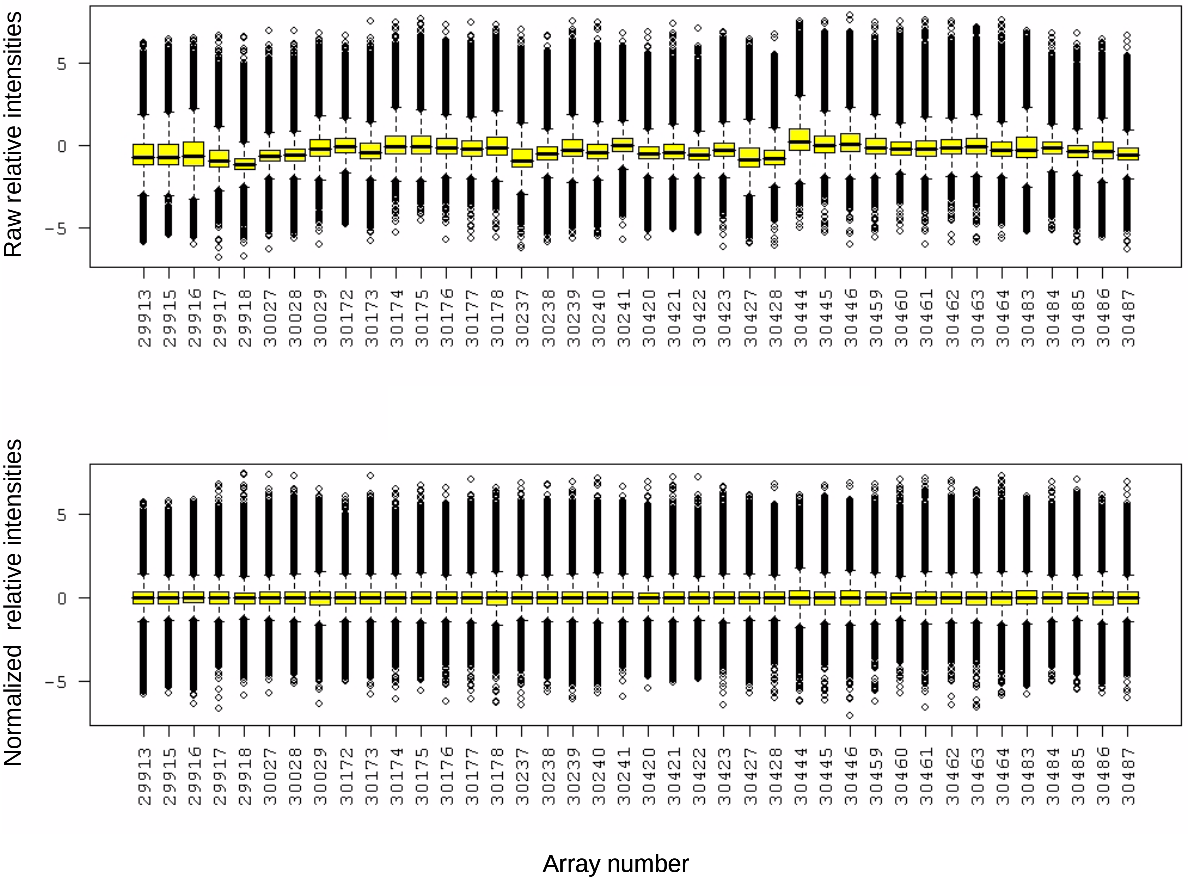

Raw relative intensities
5
0
−5
29913 29915 29916 29917 29918 30027 30028 30029 30172 30173 30174 30175 30176 30177 30178 30237 30238 30239 30240 30241 30420 30421 30422 30423 30427 30428 30444 30445 30446 30459 30460 30461 30462 30463 30464 30483 30484 30485 30486 30487
Normalized relative intensities
5
0
−5
29913 29915 29916 29917 29918 30027 30028 30029 30172 30173 30174 30175 30176 30177 30178 30237 30238 30239 30240 30241 30420 30421 30422 30423 30427 30428 30444 30445 30446 30459 30460 30461 30462 30463 30464 30483 30484 30485 30486 30487
Array number
